# Further studies on water mites from Korea, with description of two new species (Acari, Hydrachnidia)

**DOI:** 10.3897/zookeys.507.9973

**Published:** 2015-06-08

**Authors:** Vladimir Pešić, Ksenia A. Semenchenko, Wonchoel Lee

**Affiliations:** 1 Department of Biology, University of Montenegro, Cetinjski put b.b., 81000 Podgorica, Montenegro; 2 Institute of Biology and Soil Science, Far Eastern Branch of Russian Academy of Sciences, Vladivostok, 690022 Russia; 3 Hanyang University, Department of Life Sciences, Seoul 133-791, Korea

**Keywords:** Acari, Hydrachnidia, new species, new records, running waters

## Abstract

New records of water mites (Acari: Hydrachnidia) from streams in South Korea are presented. Two species are described as new to science: *Torrenticola neodentifera* **sp. n.** (Torrenticolidae) and *Atractides ermilovi* **sp. n.** (Hygrobatidae). Five species are reported as first records for Korea: Wandesia (Wandesia) reducta Tuzovskij, 1987, Wandesia (Wandesia) cf. rara Tuzovskij, 1990, Sperchon (Sperchon) orientalis Tuzovskij, 1990, Feltria (Feltria) kuluensis Tuzovskij, 1988 and Atractides (Atractides) constrictus (Sokolow, 1934). The latter species is redescribed and elevated to species rank based on new material from the Russian Far East.

## Introduction

Recently, the senior author (Pešić 2014) published a checklist of water mites from Korea, listing 74 species, in 32 genera and 13 families. However, the water mite fauna of Korea is still insufficiently known. This is one of the limnic groups of invertebrates for which studies have been published only very recently, as the first paper on water mites in South Korea was published as late as the last decade of the 20th century (Chung and Kim 1991). For a full bibliography see Pešić (2014).

The water mites of this study mainly were collected by the senior author during his trip in South Korea in May, 2013. The paper aims to describe this material in order to prepare the way towards the production of an identification key of this important group of freshwater invertebrates.

## Material and methods

Water mite were collected by hand netting, sorted on the spot from the living material, fixed in Koenike-fluid and dissected as described elsewhere (e.g., Gerecke et al. 2007). Holotype and paratypes of the new species will be deposited in the National Institute of Biological Resources, Korea (NIBR); material from the Russian Far East is deposited in the research collections of the Institute of Biology and Soil Science, Vladivostok, Russia (IBSS).

In the section ‘Material examined’ collecting site abbreviations derive from the geographical database Pešić. The composition of the material is given as: males/females/deutonymphs. All measurements are given in μm. For a detailed description and discussion of the characteristics of the genus *Atractides* and a detailed methodological introduction, see Gerecke (2003).

The following abbreviations are used: Ac-1 = first acetabulum, asl = above sea level, Cx-I = first coxae, Cxgl-4 = coxoglandularia of fourth coxae, Dgl-1-4 = dorsoglandularia, dL = dorsal length, H = height, L = length, Lgl-1-4 = lateroglandularia, I-L-6 = Leg 1, sixth segment (tarsus), mL = medial length, n = number of specimens examined, NP = National Park, P-1 = palp, first segment, Preoc. = preoculare; pregen = pregenital sclerite, Postoc. = postoculare; S-1 = proximal large ventral seta at I-L-5, S-2 = distal large ventral seta at I-L-5, Vgl-1 = ventroglandularia 1, vL = ventral length, W = width.

## Systematic part

### Family Hydryphantidae Piersig, 1896 Subfamily Wandesiinae Schwoerbel, 1961

#### Genus *Wandesia* Schechtel, 1912

##### Wandesia (Wandesia) reducta

Taxon classificationAnimaliaTrombidiformesHydryphantidae

Tuzovskij, 1987

Fig. 1

Wandesia reducta Tuzovskij 1987: 39. Synonymy.

###### Material examined.

SOUTH KOREA: CR22 Gangwon Province, Chiaksan NP, Silim town, stream shaded, substrate: stones, gravels; 37°17.081'N, 128°15.389'E, 25.v.2013 Pešić & Karanović 0/1/0 (mounted).

###### Remarks.

The single female from this study matches the general morphology of *Wandesia reducta* Tuzovskij, 1987. This species was described by Tuzovskij (1987) from Magadan region in the Russian Far East, based on three females and a larva. Here we give measurements of the specimen from Korea. Idiosoma L 1800, Cx-I+II total L 138, W 113, Cx-III+IV lateral L 131; Ac-1 L 31, L/W 1.5, Ac-2 L 34 L/W 1.5, Ac-3 L 43, L/W 2.2; gnathosoma vL 146, chelicera total L 206, H 42, L/H ratio 5.0, basal segment L 154, claw L 59, L basal segment/claw ratio 2.6; palp: total L 248, dL/H, dL/H ratio: P-1, 20/34, 0.59; P-2, 60/40, 1.5; P-3, 55/42, 1.3; P-4, 91/26, 3.56; P-5, 22/12, 1.9; length P-2/P-4 ratio 0.66; dL of I-L: 44, 75, 91, 106, 109, 88.

**Figure 1.**
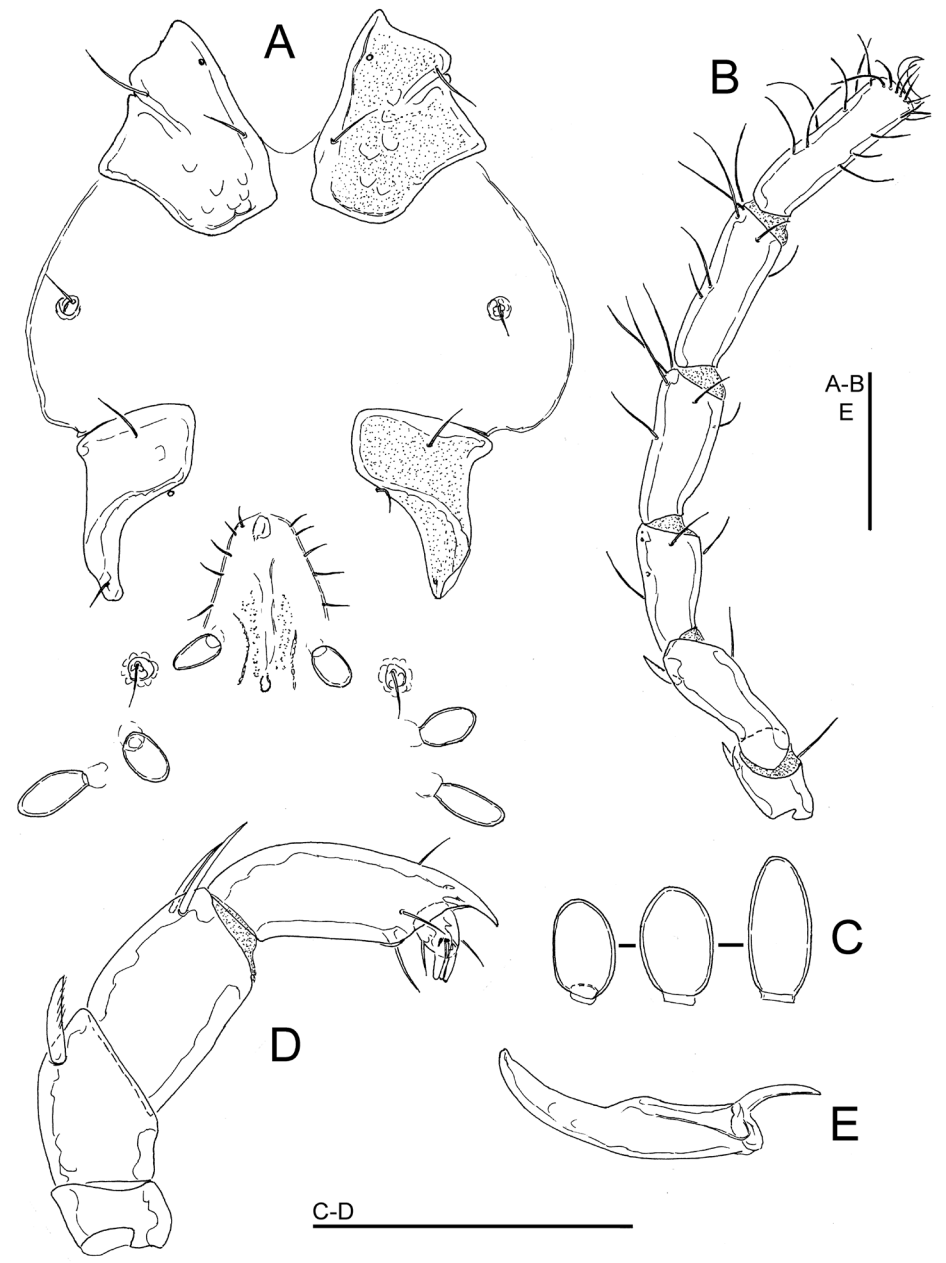
*Wandesia reducta* Tuzovskij, 1987, female, Chiaksan NP, Korea: **A** coxal and genital field **B** I-Leg **C** acetabula (Ac-1-3, from left to right) **D** palp, medial view **E** chelicera. Scale bars = 100 μm.

###### Distribution.

Far East of Russia (Tuzovskij 1987; Semenchenko 2010). New for the fauna of Korea.

##### Wandesia (Wandesia) cf. rara

Taxon classificationAnimaliaTrombidiformesHydryphantidae

Tuzovskij, 1990

Fig. 2

Wandesia rara Tuzovskij 1990: 67. Synonymy.

###### Material examined.

SOUTH KOREA: CR16 Gyeongsangbuk Province, Juwangsan NP, Woroe-ri, Cheong song-eup, Dalgikpo, waterfall, 36°26.499'N, 129°08.114'E, 23.v.2013 Pešić & Karanović 0/1/0 (mounted).

###### Morphology.

**Figure 2.**
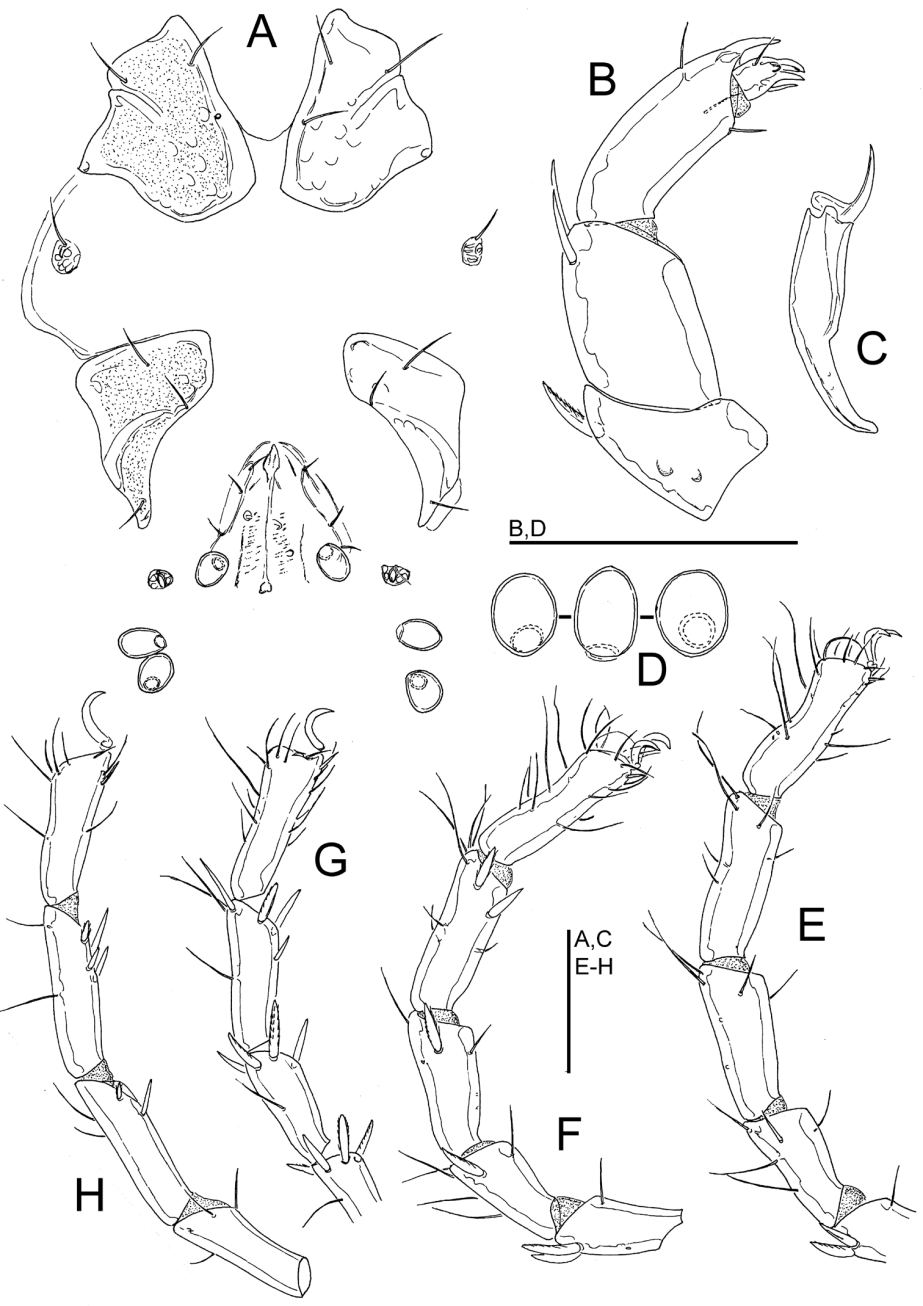
Wandesia cf. rara Tuzovskij, 1990, female, Juwangsan NP, Korea: **A** coxal and genital field **B** palp, lateral view (P-1 lacking) **C** chelicera **D** acetabula (Ac-1-3, from left to right) **E** I-L-3-6 **F** II-L-2-6 **G** III-L-4-6 **H** IV-L-3-6. Scale bars = 100 μm.

Idiosoma L 1800, coxae and genital field: Fig. 2A, Cx-I+II total L 144-146, W 111-125, Cx-III+IV lateral L 144-145; number of coxal setae: Cx-I, 3, Cx-II, 0, Cx-III, 1, Cx-IV, 1; genital field with three pairs of Ac and three setae on a transparent sclerotized strip on each side; gonopore L 106; Ac-1 L 29, L/W 1.28, Ac-2 L 32, L/W 1.5, Ac-3 L 30, L/W 1.2.

Gnathosoma vL 189, chelicera (Fig. 2C) total L 223, H 39, L/H ratio 5.8, basal segment L 150, claw L 60, L basal segment/claw ratio 2.5. Palp: dL/H, dL/H ratio: P-2, 63/41.5, 1.5; P-3, 56/45, 1.26; P-4, 94/27, 3.5; P-5, 25/12, 2.1; L P-2/P-4 ratio 0.67; palp setation as given in Fig. 2B.

Legs: setation as given in Fig. 2E–H; dorsal margin of I-L-6 and II-L-6 strongly concave (Fig. 2E–F); dL of I-L-3-6: 95, 118, 123, 123; II-L-3-6: 92, 111, 120, 122; III-L-3-6: 71, 191, 109, 121; IV-L-3-6: 108, 129, 122, 123.

###### Remarks.

With regard to similar setation of coxae, P-5 without a flagelar seta and dorsal margin of the tarsus of I-L and II-L strongly concave, the new species resembles *Wandesia rara* Tuzovskij, 1990 a species described from the Primory Territory in the Russian Far East on the basis of a single deutonymph (Tuzovskij 1990). The difference is found in more elongated Ac (L/W 2.0, calculated from figure 38-1, of Tuzovskij (1990)) in the deutonymph of *Wandesia rara*. Thus, our assignment of specimen from Korea is tentative. Only with more material in the future, and finding of a adults from the *locus typicus* it will be possible to decide whether the specimen from Korea is conspecific with *Wandesia rara* or a species new for science.

###### Distribution.

Far East of Russia (Primory Territory – Tuzovskij 1990). New for the fauna of Korea.

### Family Sperchontidae Thor, 1900

#### Genus *Sperchon* Kramer, 1877

##### Sperchon (Sperchon) orientalis

Taxon classificationAnimaliaTrombidiformesSperchonidae

Tuzovskij, 1990

Fig. 3

Sperchon orientalis Tuzovskij 1990: 99. Synonymy.

###### Material examined.

SOUTH KOREA: CR20 Chungcheongbuk Province, Mt. Vorak, Deokjusanseong, stream, 36°51.705'N, 128°06.030'E, 25.v.2013 Pešić & Karanović 2/1/0 (1/0/0 mounted).

###### Remarks.

**Figure 3.**
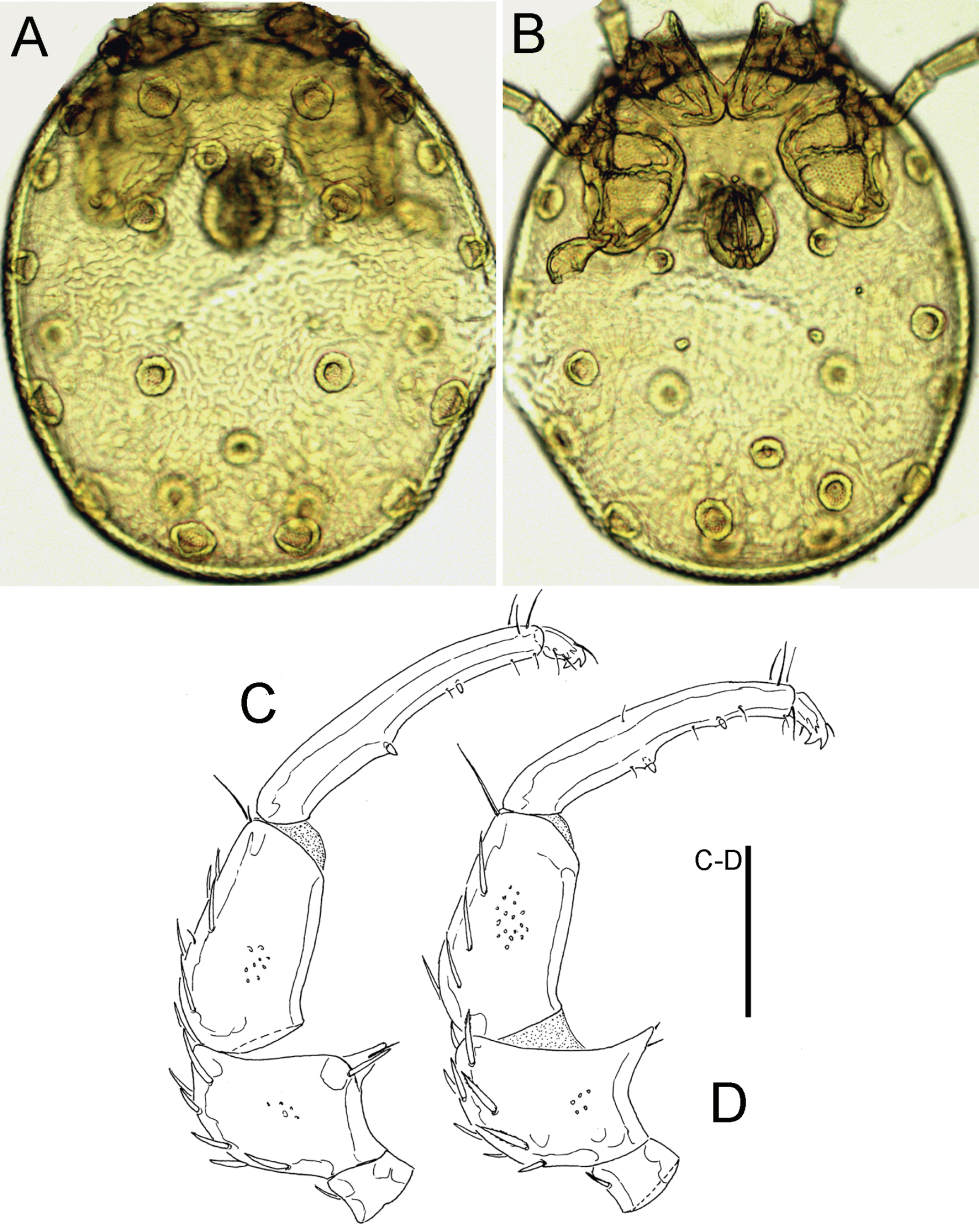
*Sperchon orientalis* Tuzovskij, 1990, male, Deokjusanseong, Korea (**A–B** photograph, **C–F** line drawing): **A** idiosoma, dorsal view **B** idiosoma, ventral view **C** palp, lateral view **D** palp, medial view. Scale bar = 100 μm (**C–D**).

The specimens examined from South Korea matches the general morphology of *Sperchon orientalis* Tuzovskij, 1990, a species described from the Primory Territory in the Russian Far East (Tuzovskij 2008). Due to the general shape of idiosoma (Cx-I medially separated, excretory pore surrounded by a sclerotized ring, see Fig. 3B) and palp (P-4 ventral setae strongly developed and projecting, dividing this segment in three equal parts in size, Fig. 3C–D), *Sperchon orientalis* closely resembles *Sperchon glandulosus* Koenike, 1886, from which it differs by the eye capsule longer than diameter of Postoc., a higher number of dorsal setae on P-2 and -3 and less densely arranged dorsal setae on IV-L-3-5 (Tuzovskij 2008).

Chung and Kim (1991) reported and illustrated *Sperchon fluviatilis* Uchida, 1934 from Korea. However, the excretory pore in *Sperchon fluviatilis* is smooth (see Uchida 1934, fig. 12–13), not sclerotized as in the illustrated male specimen from Korea. As their illustrations (Chung and Kim 1991: fig. 3A–D) show a general conformity with *Sperchon orientalis*, it is likely that the specimens attributed to *Sperchon fluviatilis* refer to *Sperchon orientalis*.

###### Distribution.

Far East of Russia, eastern Siberia (Tuzovskij 2008; Semenchenko et al. 2010). New for the fauna of Korea.

### Family Torrenticolidae Piersig, 1902 Subfamily Torrenticolinae Piersig, 1902

#### Genus *Torrenticola* Piersig, 1896

##### Torrenticola (Torrenticola) neodentifera sp. n.

Taxon classificationAnimaliaTrombidiformesTorrenticolidae

http://zoobank.org/094C3931-DDEF-4E80-83F0-8A6C1F55BFB4

Figs 4 , 5 , 6

Torrenticola dentifera Pešić et al. 2013: 25, figs 2, 7B. Synonymy.

###### Type series.

Holotype male (NIBR), dissected and slide mounted, SOUTH KOREA: CR21 Chungcheongbuk Province, Woraksan NP, Jungseonam, River exposed to sunlight, sand, gravel substrate, 36°52.644'N, 128°17.784'E, 25.v.2013 Pešić & Karanović. Paratype (NIBR): one female, CR22 Gangwon Province, Chiaksan NP, Silim town, stream shaded, stones, gravels, 37°17.081'N, 128°15.389'E, 25.v.2013 Pešić & Karanović, dissected and slide mounted.

###### Diagnosis.

Idiosoma dimensions relatively large (L 580-630); dorsal shield with colour pattern as illustrated in Fig. 5A; P–2 with a laterally compressed, anteriorly directed ventrodistal extension; P-3 with a broad, subrectangular ventrodistal projection.

###### Description.

**Figure 4.**
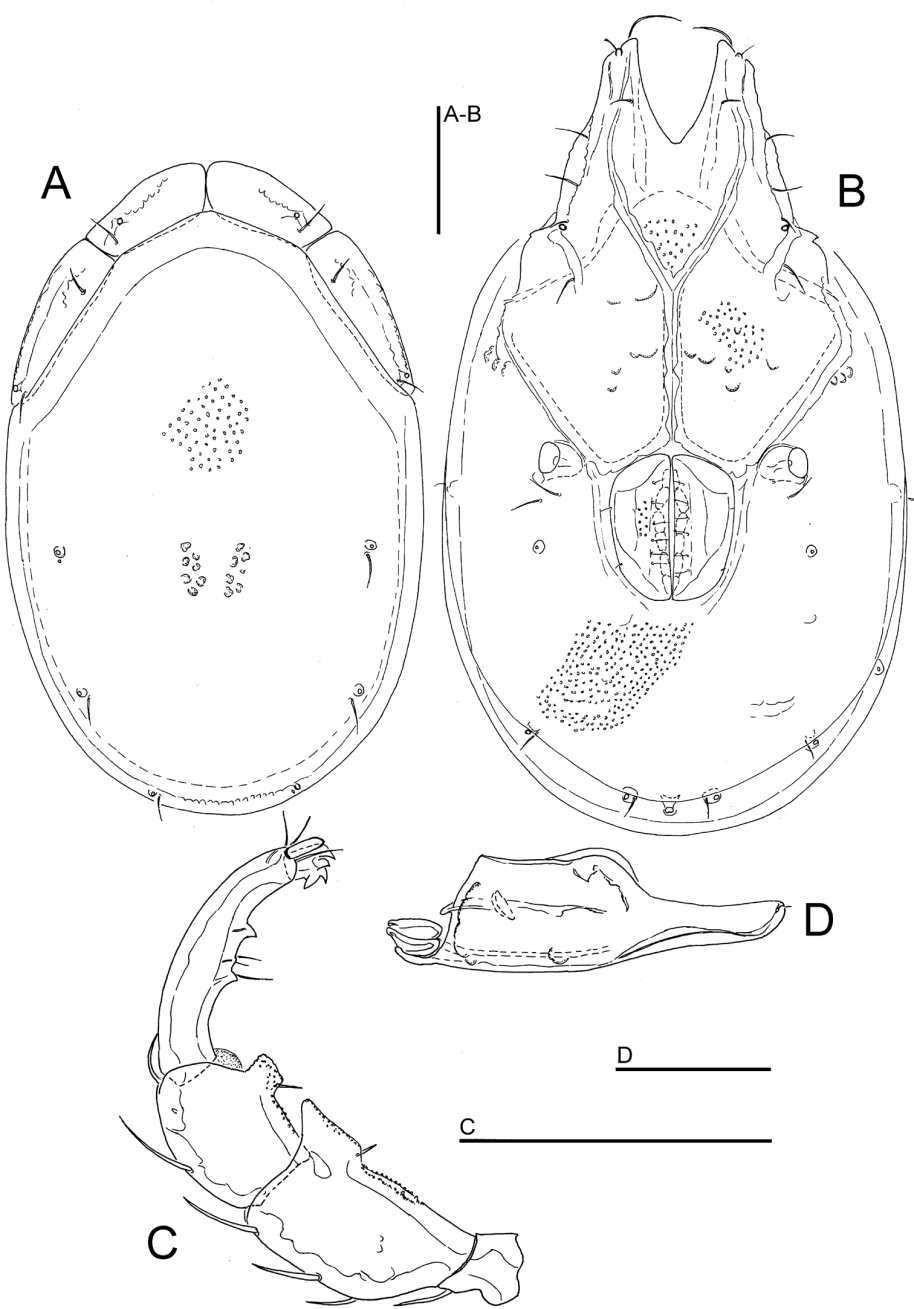
*Torrenticola neodentifera* sp. n., male holotype, Woraksan NP, Korea: **A** dorsal shield **B** ventral shield **C** palp, medial view **D** gnathosoma. Scale bars = 100 μm.

**Figure 5.**
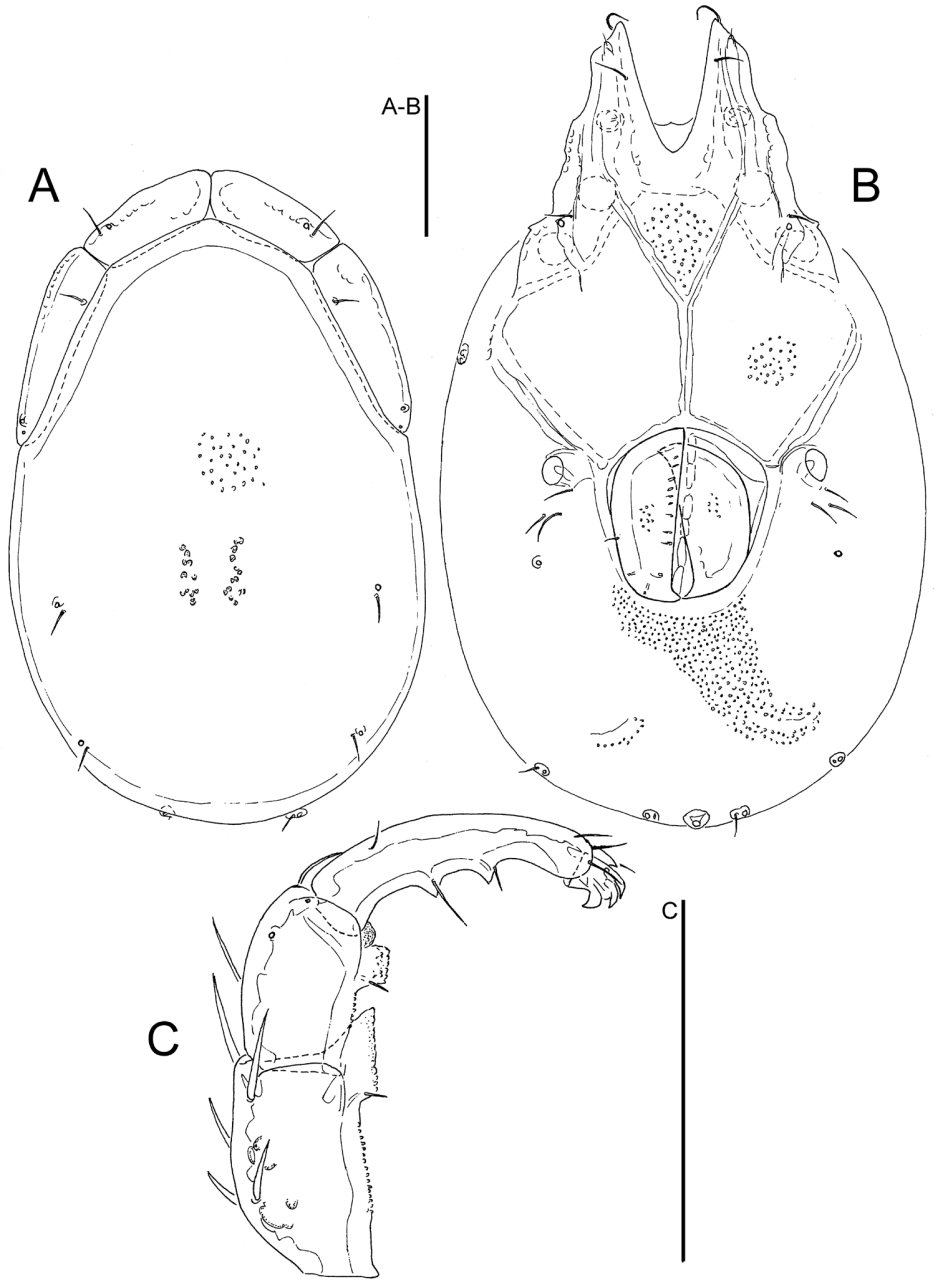
*Torrenticola neodentifera* sp. n., female, Chiaksan NP, Korea: **A** dorsal shield **B** ventral shield **C** palp, lateral view. Scale bars = 100 μm.

**Figure 6.**
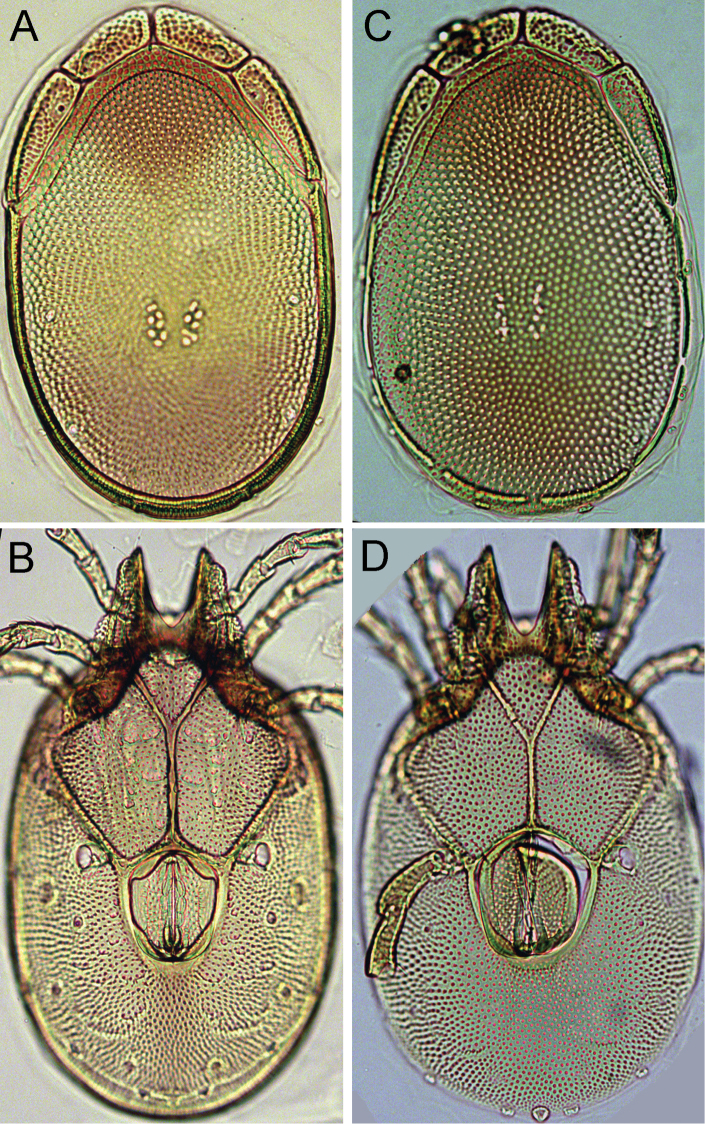
Photographs of *Torrenticola neodentifera* sp. n. (**A–B** male holotype, **C–D** female paratype): **A, C** = dorsal shield **B, D** = ventral shield.

*General features* — Idiosoma elongated; dorsal shield with colour pattern as illustrated in Fig. 6A,C; frontal platelets anteriorly bulging (Figs 4A, 5A); gnathosomal bay V-shaped; Cxgl-4 subapical, only slightly posterior of Cx-I tips; medial suture line of Cx-II+III long; posterior suture line of Cx-IV in its medial part perpendicular to the longitudinal body axis, laterally distinctly curving anteriorly; excretory pore and Vgl-2 on the line of primary sclerotization near posterior idiosoma margin; gnathosoma ventral margin only slightly curved, rostrum well developed; P-2 shorter than P-4, ventral margin of P-2 with a fine denticulation also in proximal half of the segment, distally with a laterally compressed, anteriorly directed hyaline extension and a very short, denticle-like seta laterally at base of projection; P-3 with a broad, subrectangular, apically serrated ventrodistal projection with a fine denticles, and a short seta laterally at base of projection; P-4 ventral tubercles well developed and separated (Figs 4C, 5C). *Male*. Genital field subrectangular; ejaculatory complex conventional in shape (Fig. 2D in Pešić et al. 2013). *Female*. The short postgenital area and caudal position of the excretory pore (Fig. 5B) in the specimen from Korea are due to the obviously juvenile age (indicated by weak sclerotization and absence of eggs); genital field pentagonal in shape.

*Measurements*. *Male* — Idiosoma (ventral view: Figs 4B, 6B) L 628, W 365; dorsal shield (Figs 4A, 6A) L 516, W 331, L/W ratio 1.56; dorsal plate L 477; shoulder platelet L 141-143, W 41-44, L/W ratio 3.2-3.5; frontal platelet L 106-109, W 46-47, L/W ratio 2.3; shoulder/frontal platelet L ratio 1.29-1.35. Gnathosomal bay L 81, Cx-I total L 200, Cx-I mL 118, Cx-II+III mL 127; ratio Cx-I L/Cx-II+III mL 1.58; Cx-I mL/Cx-II+III mL 0.93. Genital field L/W 116/98, ratio 1.18; ejaculatory complex L 162; distance genital field-excretory pore 162, distance genital field-caudal margin 184. Gnathosoma vL 256; chelicera total L 270; palp: total L 239, dL/H, dL/H ratio: P-1, 22/21, 1.05; P-2, 65/39, 1.7; P-3, 49/35, 1.4; P-4, 86/19, 4.6; P-5, 17/11, 1.6; P-2/P-4 ratio 0.76.

*Female* — Idiosoma (ventral view: Figs 5B, 6D) L 581, W 353; dorsal shield (Figs 5A, 6C) L 472, W 303, L/W ratio 1.56; dorsal plate L 439; shoulder platelet L 144-150, W 38-42, L/W ratio 3.4-4.0; frontal platelet L 103-105, W 44-56, L/W ratio 1.8-2.4; shoulder/frontal platelet L ratio 1.37-1.46. Gnathosomal bay L 94, Cx-I total L 206, Cx-I mL 110, Cx-II+III mL 87; ratio Cx-I L/Cx-II+III mL 2.37; Cx-I mL/Cx-II+III mL 1.26. Genital field L/W 116/126, ratio 0.92; distance genital field-excretory pore 150. Gnathosoma vL 252; chelicera total L 258-262; palp: total L 225, dL/H, dL/H ratio: P-1, 19/20, 0.95; P-2, 63/37, 1.7; P-3, 49/32, 1.54; P-4, 79/20, 4.0; P-5, 15/10, 1.5; P-2/P-4 ratio 0.8.

###### Etymology.

Named for its similarity with *Torrenticola dentifera* Wiles, 1991.

###### Remarks.

Pešić et al. (2013) collected a single male from a stream in Naebyeansan National Park, South Korea and assigned it to *Torrenticola dentifera*. This specimen is in perfect agreement with specimens examined in our study. In the original description of *Torrenticola dentifera*, a species described on the basis of two males from Selangor, Peninsular Malaysia (Wiles 1991), no information on colour pattern of dorsal shield and shape of ejaculatory complex are given. At the present state of art, males of *Torrenticola dentifera* can be distinguished from the new species by smaller dimensions of idiosoma and palps, and a more slender ventrodistal projection on P-3.

###### Habitat.

A permanent sandy/bouldary stream, shaded by riparian vegetation (Fig. 13C–D).

###### Distribution.

Korea (“*Torrenticola dentifera*“ Pešić et al. 2013, this study).

### Family Hygrobatidae Koch, 1842

#### Genus *Hygrobates* Koch, 1837

##### Hygrobates (Rivobates) cf. microepimeratus

Taxon classificationAnimaliaTrombidiformesHygrobatidae

(Sokolow, 1934)

Fig. 7

Rivobates microepimeratus Sokolow 1934: 356. Synonymy.

###### Material examined.

SOUTH KOREA: CR20 Chungcheongbuk Province, Mt. Vorak, Deokjusanseong, stream, 36°51.705'N, 128°06.030'E, 25.v.2013 Pešić & Karanović 0/1/0 (mounted).

###### Remarks.

The single female from this study matches the general morphology of *Hygrobates microepimeratus* (Sokolow, 1934) a species described from the Primory Territory in the Russian Far East (Sokolow 1934), and later on reported by Chung and Kim (1997) from Korea. This species is known from a female only making it difficult to separate from other similar species, i.e. *Hygrobates ezoensis* Uchida, 1934 (Russia: Sakhalin, Japan: Hokkaido) and *Hygrobates taniguchii* Imamura, 1954 (Japan, Hokkaido). In the original description Sokolow (1934, 1940), in addition to more slender P-3 (compared with *Hygrobates diversiporus*), gave particular weight to the smaller dimensions of coxae which occupy one third of venter. According to Imamura (1954) *Hygrobates taniguchii* differs from *Hygrobates microepimeratus* in larger coxae. *Hygrobates ezoensis* differs in P-2 with a more pronounced and acute ventrodistal projection and stouter P-3 (see Matsumoto et al. 2005).

**Figure 7.**
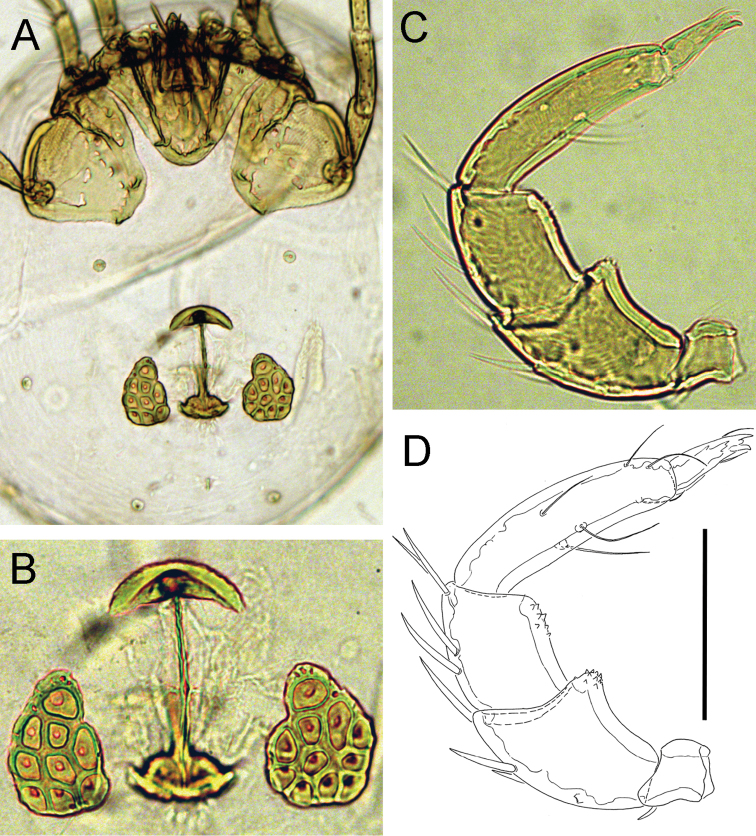
Hygrobates (Rivobates) cf. microepimeratus (Sokolow, 1934), female, Deokjusanseong, Korea (**A–C** photographs, **D** line drawing): **A** idiosoma, ventral view **B** genital field **C–D** palp. Scale bar = 100 μm (**D**).

###### Distribution.

Far East of Russia (Primory Territory – Sokolow 1934), Korea (Chung and Kim 1997, this study).

#### Genus *Atractides* Koch, 1837

##### Atractides (Atractides) constrictus

Taxon classificationAnimaliaTrombidiformesHygrobatidae

(Sokolow, 1934) stat. n.

Figs 8 , 9 , 10

Megapus nodipalpis constrictus Sokolow 1934: 361. Synonymy.

###### Material examined.

SOUTH KOREA: CR22 Gangwon Province, Chiaksan NP, Silim town, stream (shaded, stones and gravel substrate), 37°17.081'N, 128°15.389'E, 25.v.2013 Pešić & Karanović 5/0/0 (1/0/0 mounted). RUSSIA: Primory Territory, Khasansky District, “Kedrovaya Pad National Nature Biosphere Reserve”, Sea of Japan basin, Kedrovaya River (depth 12–50 cm; substrate: boulders, cobbles, pebbles), 43°06.056'N; 131°33.310'E; 27.vi.1993 Tiunova 4/15/0 (2/2/0 mounted); Primory Territory, Partizansky District, Partizanskay River basin, Tigrovaya River (substrate: cobbles, pebbles, sand), 43°11.401'N; 133°12.660'E, 12.vi.2010 Semenchenko & Sidorov 2/3/2 (1/1/2 mounted).

###### General features.

**Figure 8.**
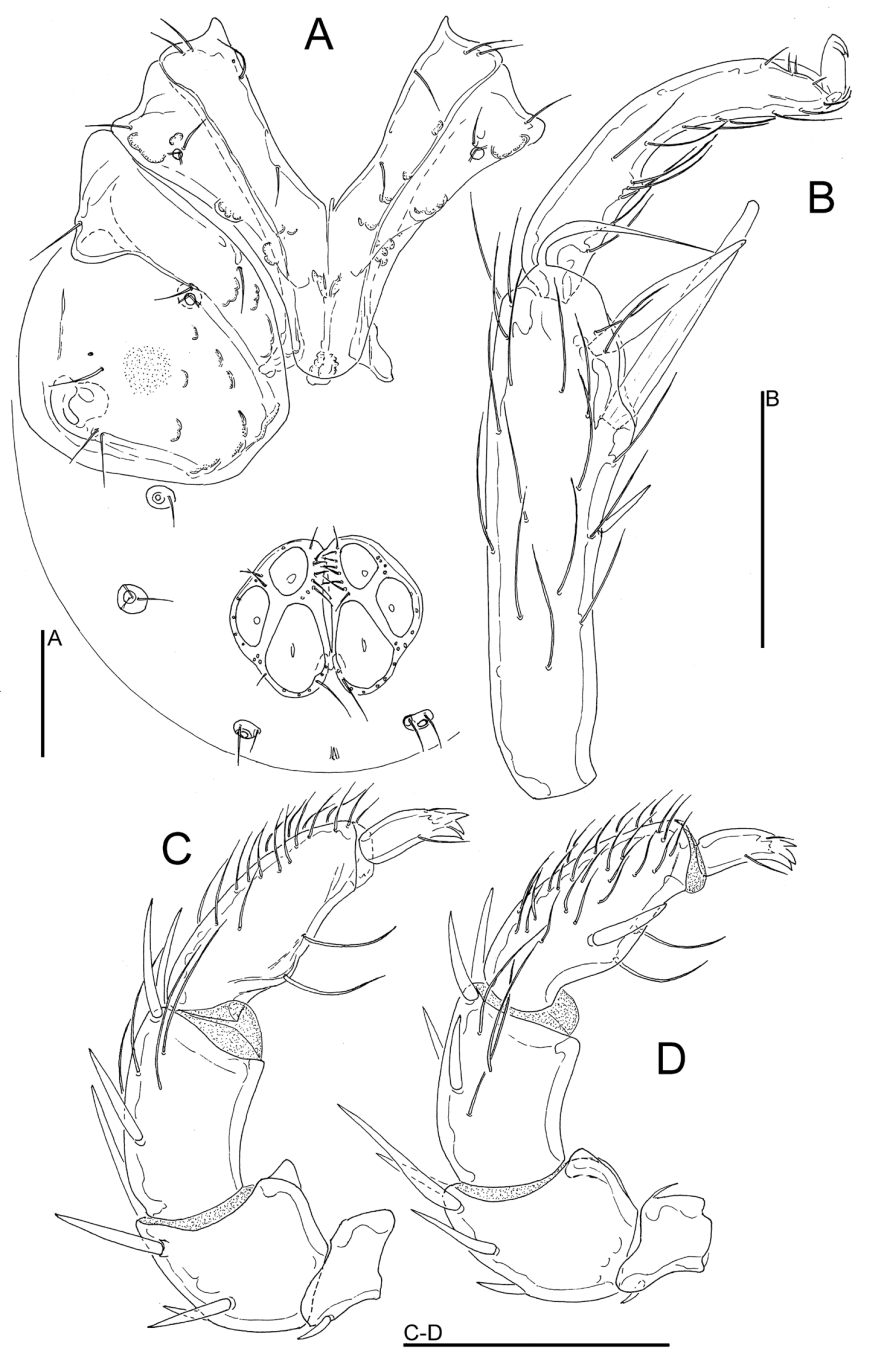
*Atractides constrictus* (Sokolow, 1934), male, Chiaksan NP, Korea: **A** idiosoma, ventral view **B** I-L-5 and -6 **C** palp, lateral view **D** palp, medial view. Scale bars = 100 μm.

**Figure 9.**
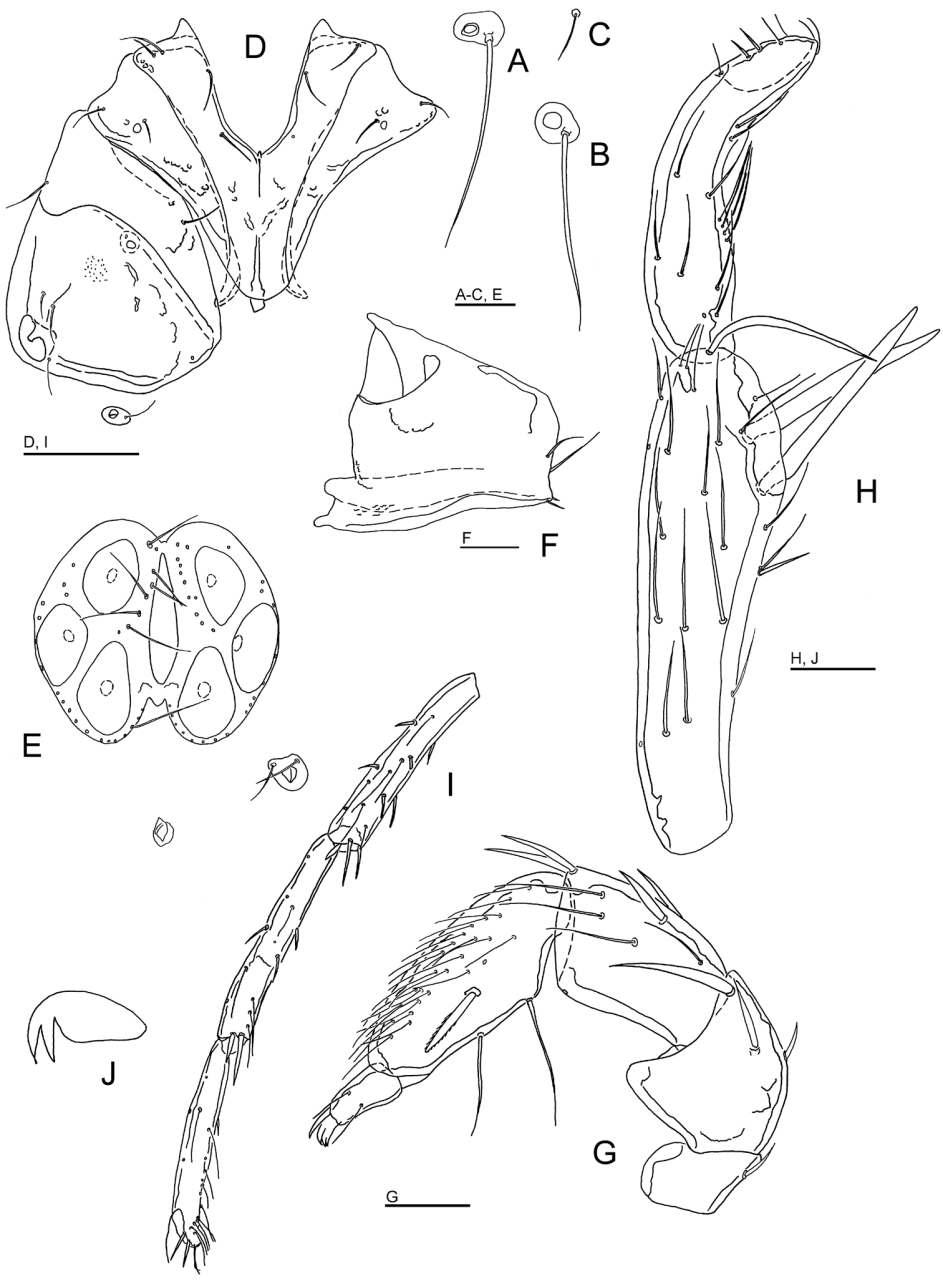
*Atractides constrictus* (Sokolow, 1934), male, Tigrovaya River, Russia: **A** seta Dgl-1 **B** seta Dgl-4 **C** Postoc. **D** coxal field **E** genital field, excretory pore and Vgl-1 fused to Vgl-2 **F** gnathosoma **G** palp, medial view **H** I-L-5 and -6 **I** IV-L-4-6 **J** legs claw. Scale bars = 100 μm (**D, I**), 25 μm (**A–C, E–H, J**).

**Figure 10.**
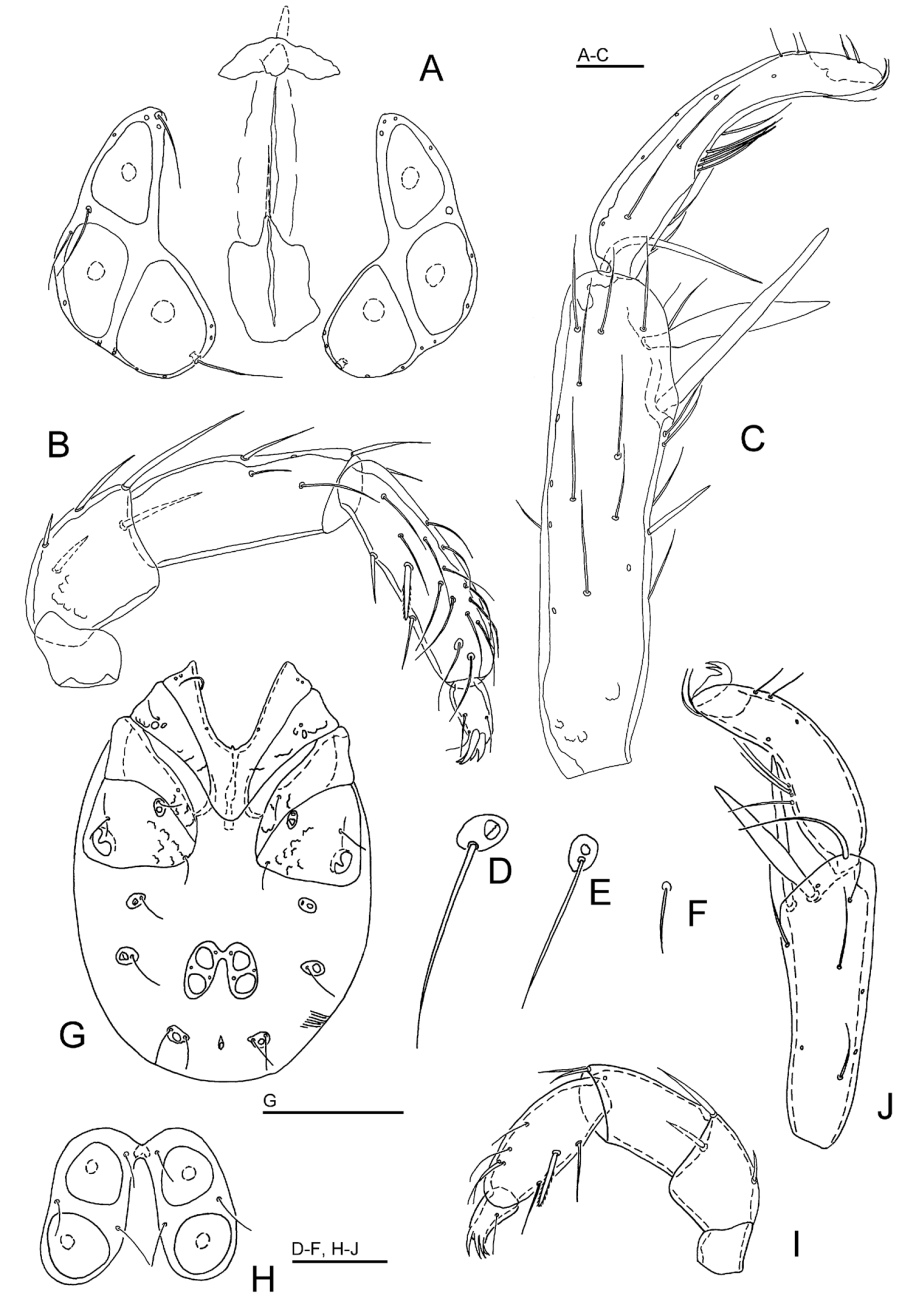
*Atractides constrictus* (Sokolow, 1934), Tigrovaya River, Russia (**A–C** female, **D–J** deutonymph): **A** genital field **B** palp, medial view **C** I-L-5 and -6 **D** seta Dgl-1 **E** seta Dgl-4 **F** Postoc. **G** idiosoma, ventral view **H** genital field **I** palp, medial view **J** I-L-5 and -6. Scale bars = 100 μm (**G**), 25 μm (**A–F, H–J**).

*Adults*. Integument striated, muscle insertions unsclerotized. Setae Dgl-1, Dgl-3, Dgl-4 and Lgl-2 longer than other dorsal setae; Preoc. and Postoc. without glandularia (Fig. 9A–C). Coxal field: caudal margin Cx-I convex, apodemes of Cx-II directed laterally (Figs 8A, 9D). Excretory pore smooth; Vgl-1 fused to Vgl-2, separate in juvenile specimens. Gnathosoma elongated (Figs 9F). Palp with strong sexual dimorphism in P-2 and -4, P-4 sword seta between ventral setae. Legs without swimming setae, posterior legs slender (Fig. 9I). Leg claws with near-equal denticles (Fig. 9J). I-L-5: S-1 longish, blunt, S-2 basally enlarged, pointed; I-L-6 curved, basally thickened (Figs 8B, 9H, 10C). *Male*. Genital field: both anterior and posterior margins deeply indented, Ac in a triangle, Ac-3 strongly enlarged (Figs 8A, 9E). Palp: P-2 with strong ventrodistal protrusion consisting of a bluntly pointed medial hump and a convex lateral thickening; P-3 ventral margin concave; P-4 maximum H near proximoventral seta, sword seta between ventral setae (Figs 8C–D, 9G). *Female*. Ac arranged in an obtuse angle (Fig. 10A). Palp: P-2 ventrodistal edge rounded; P-3 ventral margin straight or slightly concave; P-4 slender, slightly protruding near proximoventral seta (Fig. 10B).

*Deutonymphs*. Setae Dgl-1, Dgl-3, Dgl-4 and Lgl-2 longer than other dorsal setae; Preoc. and Postoc. without glandularia (Figs 10D–F). Coxal field: covers about one half of ventral surface. Caudal margin of Cx-I convex. Excretory pore smooth; Vgl-1 fused to Vgl-2 (Fig. 10G). Genital field: 2 pairs of acetabula placed on fused anteriorly genital plates, each bearing 3 thin setae (Fig. 10H). Palps: P-2 with 1 proximal and 2 distal setae; P-3 with 2 distal setae; P-4 slightly convex, sword seta between ventral setae (Fig. 10I). Setation on I-L-5-6 similar to adults (Fig. 10J).

*Measurements*. *Male* (from CR22, in parentheses specimen from Russia, Tigrovaya River) — Idiosoma L/W 600/538 (560/476); coxal field: L 369 (336); Cx-III W 409 (383); Cx-I+II mL 138 (135), lL 263 (238); genital field L/W 129 (109)/ 157 (122), L Ac 1-3: 40-42 (37-42), 51-52 (39-40), 72 (50-51); ejaculatory complex L 127 (106).

Palp: Total L 317 (275), dL/H: P-1, 29/34 (29/36, 0.81); P-2, 77/55 (65/56); P-3, 75/48 (56/46); P-4, 97/29 (93/38); P-5, 39/15 (32/16); L P-2/P-4 ratio 0.79 (0.69). Gnathosoma vL 153 (127).

Legs: I-L-5 dL 218 (174), vL 142 (151), dL/vL ratio 1.53 (1.44), maximum H 59 (49), dL/maximum H 3.7 (4.45), S-1 L 105 (84), L/W ratio 8.5 (12), S-2 L 74 (73), L/W ratio 5.4 (9.1), distance S-1-2, 24 (16), L ratio S-1/2, 1.42 (1.15); I-L-6 dL 139 (120); central H 21 (20), dL/central H ratio 6.7 (6); dL I-L-5/6 ratio 1.57 (1.45).

*Female* (from Kedrovaya River, Russia, in parentheses specimen from Tigrovaya River) — Idiosoma L/W 765/730 (730/600); coxal field: L 405 (369); Cx-III W 402 (468); Cx-I+II mL 138 (139), lL 290 (264); genital field L/W 162 (142)/197 (174), genital plate L 137 (118); pregen W 65 (62); L Ac 1-3: 50 (47), 56 (50), 54 (51).

Palp: Total L 400 (368), dL/H: P-1, 38/51 (43/29); P-2, 89/76 (72/44); P-3, 116/56 (105/43); P-4, 124/35 (116/29); P-5, 33/19 (32/16); L P-2/P-4 ratio 0.72 (0.62). Gnathosoma vL 160 (147).

Legs: I-L-5 dL 248 (221), vL 170 (156), dL/vL ratio 1.46 (1.42), maximum H 79 (56), dL/maximum H 3.1 (3.9), S-1 L 124 (108), L/W ratio 12.4 (13.5), S-2 L 97 (82), L/W ratio 7.5 (8.2), distance S-1-2, 28 (24), L ratio S-1/2, 1.29 (1.32); I-L-6 dL 179 (156); central H 22 (20), dL/central H ratio 8.1 (7.8); dL I-L-5/6 ratio 1.36 (1.42).

*Deutonymph* — Idiosoma L/W 323/237); coxal field: L 173; Cx-III W 191; Cx-I+II mL 62, lL 119; genital plate L/W 49/28; L Ac 1-2: 19, 17.

Palp: Total L 165, dL/H: P-1, 18/18; P-2, 37/30; P-3, 43/22; P-4, 49/21; P-5, 18/10; L P-2/P-4 ratio 0.75. Gnathosoma vL 81.

Legs: I-L-5 dL 96, vL 76, dL/vL ratio 1.3, maximum H 31, dL/maximum H 1.5, S-1 L 47, L/W ratio 11.8, S-2 L 46, L/W ratio 9.2, distance S-1-2, 2.5, L ratio S-1/2, 1.02; I-L-6 dL 70; central H 16, dL/central H ratio 4.4; dL I-L-5/6 ratio 1.36.

###### Remarks.

*Atractides constrictus* was originally described by Sokolow (1934) from the Primory Territory in the Russian Far East as a ‘variety’ of *Atractides nodipalpis*. In the original description Sokolow (1934, 1940) gave particular weigt to the shape of P-4 in males: strongly thickened near proximoventral seta, basally strongly narrowed, ventral setae more closely approaching to each other. However, there is no reason to support the placement of this taxon as a subspecies of *Atractides nodipalpis*, as from the latter species, *Atractides constrictus* can easily be distinguished by the fused Vgl-1 and -2. Males examined from Korea show a general conformity with material from the Far East of Russia. Differences are found in a more enlarged Ac-3, a larger S-1/2 interspace and I-L-6 relatively longer compared to I-L-5 in specimens from Korea.

###### Distribution.

Far East of Russia (Primory Territory – Sokolow 1934). New for the fauna of Korea.

##### Atractides (Atractides) gracilis

Taxon classificationAnimaliaTrombidiformesHygrobatidae

(Sokolow, 1934)

Megapus gracilis Sokolow 1934: 366. Synonymy.

###### Material examined.

SOUTH KOREA: CR17 Gyeongsangbuk Province, Hupo-Myeon, shadded stream, 36°40.996'N, 129°25.201'E, 24.v.2013 Pešić & Karanović 0/1/0; CR18 Gyeongsangbuk Province, Haenggok-ri, river, exposed to sunlight, 36°57.182'N, 129°17.670'E, 24.v.2013 Pešić & Karanović 0/1/0.

###### Distribution.

Far East of Russia (Arsenyevka River basin – Sokolow 1934); Japan; Korea (Kim and Chung 1991, Pešić 2014, this study).

##### Atractides (Atractides) ermilovi sp. n.

Taxon classificationAnimaliaTrombidiformesHygrobatidae

http://zoobank.org/ABC02400-8864-4BAD-B3E8-F7AAD7B9DBDE

Fig. 11

###### Type series.

Holotype male (NIBR), dissected and slide mounted, SOUTH KOREA: CR19 Chungcheongbuk Province, Sobaeksan NP, shaded stream, 36°57.660'N, 128°25.534'E, 24.v.2013 Pešić & Karanović.

###### Diagnosis

(Female unknown). Median suture line relatively Cx-I+II long (> 100 μm); acetabula large (maximum diameter > 50 μm) in triangular position; ventrodistal protrusion of P-2 conus- shaped; S-1 distally truncated, S-2 thicker and shorter, small setal interspace (8 μm): I-L-6 short (L I-L-5/6 ratio 1.59) and stout (L/H ratio 5.0).

###### General features.

**Figure 11.**
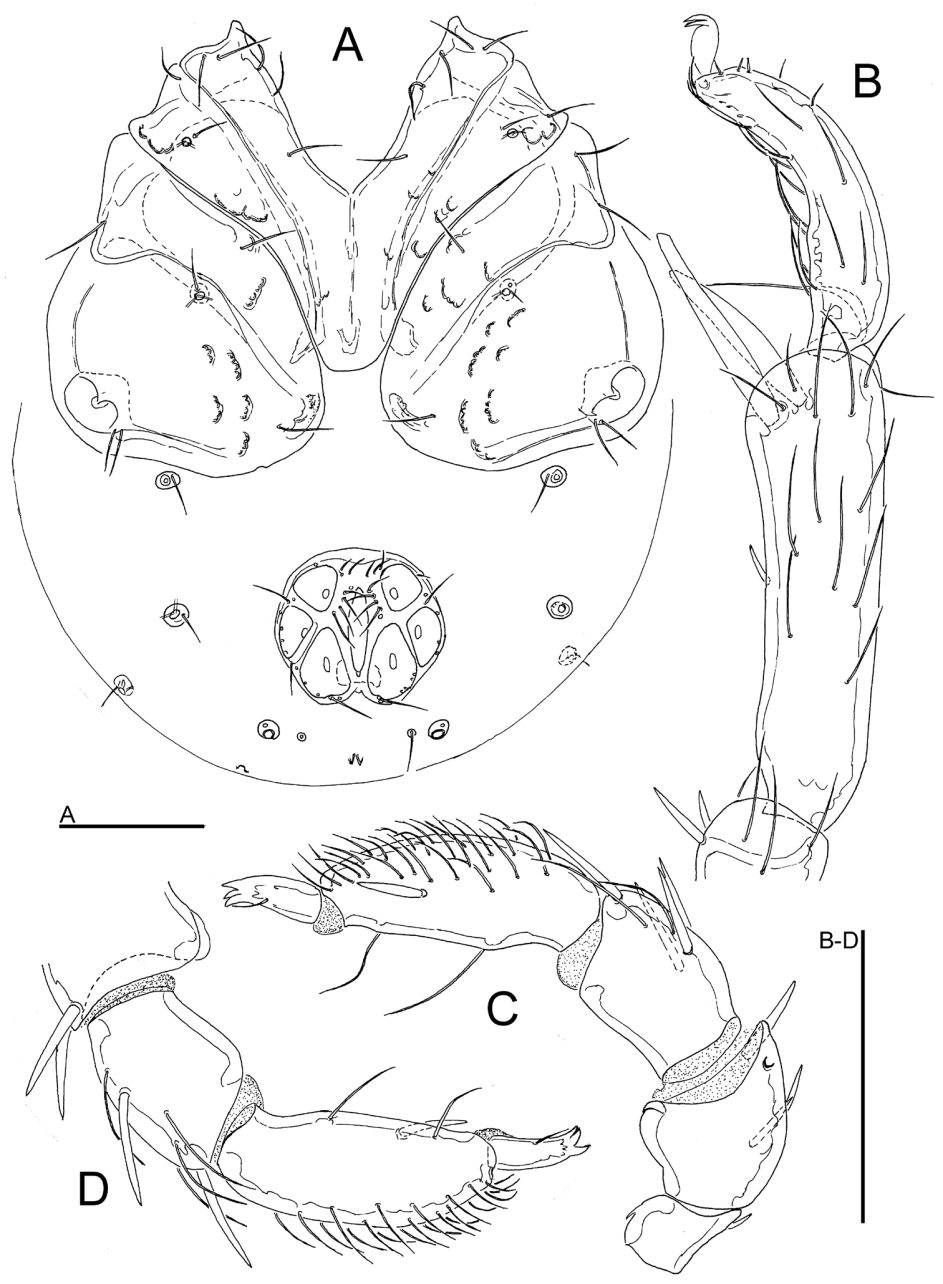
*Atractides ermilovi* sp. n., male, CR19 Sobaeksan NP, Korea: **A** idiosoma, ventral view **B** I-L-5 and -6 **C** palp, medial view **D** palp (P-3-5), lateral view. Scale bars = 100 μm.

Integument striated, muscle insertions unsclerotized. Coxal field: caudal margin Cx-I straight, apodemes of Cx-II directed laterally. Genital field: anterior margin of primary sclerotization slightly concave, but secondary sclerotization forming narrow semicircular border, posterior margin slightly indented, Ac in triangular position (Fig. 11A). Excretory pore smooth; Vgl-1 not fused to Vgl-2. Palp (Fig. 11C–D): strong ventrodistal protrusion of P-2; P-3 weakly concave proximally; P-4 sword seta between ventral setae, but approached to distoventral seta. I-L-5 (Fig. 11B): dorsal and ventral margins subparallel basally and centrally but diverging near the distal edge, S-1 and -2 close together, S-1 distally truncated, S-2 thicker and shorter, bluntly pointed; I-L-6 stout and curved, basally thickened, distally equally narrowed.

*Measurements* — Idiosoma L/W 550/425; coxal field: L 319; Cx-III W 366; Cx-I+II mL 121, lL 231; genital field L/W 112/120, L Ac 1-3: 34, 42, 51-55.

Palp: Total L 289, dL/H, dL/H ratio: P-1, 31/25, 1.25; P-2, 66/51, 1.3; P-3, 62/44, 1.39; P-4, 96/26, 3.67; P-5, 34/13, 2.5; L P-2/P-4 ratio 0.96.

Legs: I-L-5 dL 171, vL 132, dL/vL ratio 1.3, maximum H 48, dL/maximum H 3.59, S-1 L 77, L/W ratio 8.4, S-2 L 65, L/W ratio 6.0, distance S-1-2, 8, L ratio S-1/2, 1.19; I-L-6 dL 108, central H 22, dL/central H ratio 5.0; dL I-L-5/6 ratio 1.59.

*Female*: unknown.

###### Etymology.

Named after Dr Sergey Ermilov (Tyumen, Russia), for his contribution to the taxonomy of oribatid mites.

###### Remarks.

The new species resembles *Atractides samsoni* (Sokolow, 1936) in the small S-1/2 interspace, I-L-6 stocky, postgenital area with smooth excretory pore and unfused Vgl-1/2 and a palp with a conus shaped ventrodistal protrusion in male. The latter species can be distinguished by the shorter medial suture line of Cx-I, smaller acetabula, more slender S-1 and -2, and I-L-6 only weakly curved and longer (see Gerecke 2003). Males of *Atractides constrictus* (Sokolow, 1934), a species similar in the shape of palp (double ventral protrusion on P-2), a larger Ac (maximum diameter > 39 μm), and I-L-6 relatively short compared to I-L-5 (L I-L-5/6 ratio 1.4-1.6), differ in wider setal interspace on I-L-5, I-L-6 more slender and more narrow centrally, P-4 ventral setae inserted more closely to each other and genital field deeply indented both anteriorly and posteriorly.

###### Habitat.

A permanent sandy/bouldary stream, shaded by riparian vegetation (Fig. 13B).

###### Distribution.

Korea, only known from the locus typicus.

### Family Feltriidae K.Viets, 1926

#### Genus *Feltria* Koenike, 1892

##### Feltria (Feltria) kuluensis

Taxon classificationAnimaliaTrombidiformesFeltriidae

Tuzovskij, 1988

Fig. 12

Feltria kuluensis Tuzovskij 1988: 226. Synonymy.

###### Material examined.

SOUTH KOREA: CR16 Gyeongsangbuk Province, Juwangsan NP, Woroe-ri, Cheong song-eup, Dalgikpo, waterfall, 36°26.499'N, 129°08.114'E, 23.v.2013 Pešić & Karanović 1/0/0 (mounted).

###### Remarks.

**Figure 12.**
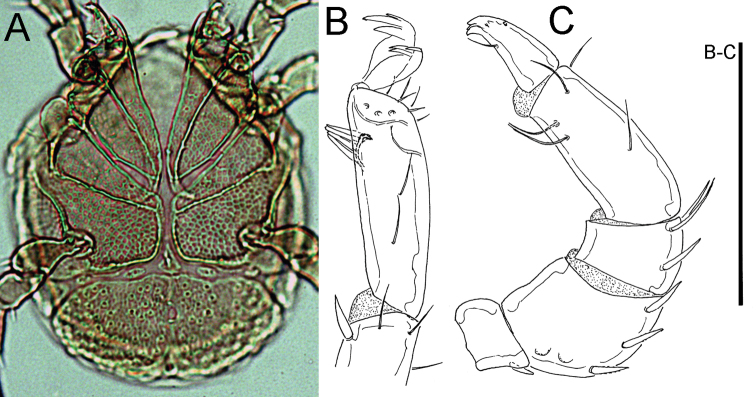
*Feltria kuluensis* Tuzovskij, 1988, male, Juwangsan NP, Korea (**A** photograph, **B–C** line drawing): **A** idiosoma, ventral view **B** IV-L-6 **C** palp. Scale bar = 100 μm (**B–C**).

The single male from this study matches the general morphology of *Feltria kuluensis* Tuzovskij, 1988, a species described from the Magadan region in the Russian Far East (Tuzovskij (1988). This species closely resembles *Feltria minuta* Koenike, 1892, a species known from central, northern and western Europe, due to the following features: dorsum in male with a large shield (including Postoc. and Dgl-1-3) and Dgl-4 on paired, transverse, laterally pointed posterodorsal platelets (fig. 7-1, in Tuzovskij 1988), genital plate in male with undulating or straight anterior margin, gonopore in central position, and more than 50 pairs of Ac scattered over the whole plate (Fig. 12A), and male IV-L-6 with a digitiform ventrolateral extension directed to distal part of segment (Fig. 12B). *Feltria minuta* differs in relatively more slender palp, P-4 with both ventral setae on slightly elevated, parallel longitudinal extensions, and male IV-L-6 relatively more shorter with ventrolateral extension bearing one fine seta and two enlarged, transparent setae, adpressed to each other and directed ventrally (Gerecke et al. 2009).

Chung and Kim (1991) reported and illustrated *Feltria ishikariensis* Imamura, 1954 from Kangreung, Korea. This species was described orginally by Uchida (1934) as *Feltria rotunda* based on three specimens (probably, by mistake assigned to a male, see figs 48–49 in Uchida 1934). Later on, Imamura (1954) described and illustrated the female of *Feltria ishikariensis* from Hokkaido, Japan. *Feltria ishikariensis* clearly differs from the species illustrated by Chung and Kim (1991) by the lower number of acetabula (< 50 pairs of Ac). As their illustrations (Chung and Kim 1991: Figs 6G–I, 7A–C) show a general conformity with *Feltria kuluensis* in all abovementioned characters, it is very likely that the specimens attributed to *Feltria ishikariensis* refer to *Feltria kuluensis*.

In the same paper, Chung and Kim (1991) assigned two female specimens collected from Kangreung to *Feltria minuta* Koenike, 1892. As mentioned by Pešić (2014), because the important characters are restricted to males, this assignment is uncertain, and probably refer to female of *Feltria kuluensis*.

###### Distribution.

Far East of Russia (Tuzovskij 1988). New for fauna of Korea.

**Figure 13.**
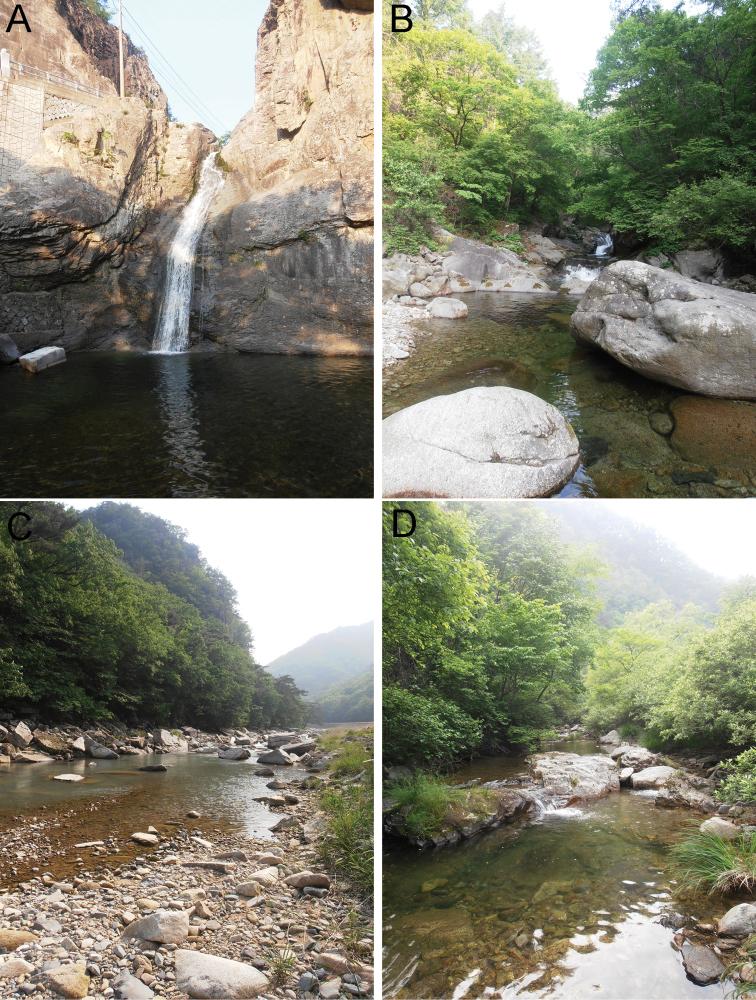
Photographs of selected sampling sites. **A** CR16 (Juwangsan NP, Dalgikpo waterfall, sampling site of Wandesia cf. rara and *Feltria kuluensis*) **B** CR19 (Sobaeksan NP, stream, type locality of *Atractides ermilovi* sp. n.) **C** CR21 (Woraksan NP, stream, type locality of *Torrenticola neodentifera* sp. n.) **D** CR22 (Chiaksan NP, stream, sampling site of *Wandesia reducta*, *Torrenticola neodentifera* sp. n. and *Atractides constrictus*).

## Supplementary Material

XML Treatment for Wandesia (Wandesia) reducta

XML Treatment for Wandesia (Wandesia) cf. rara

XML Treatment for Sperchon (Sperchon) orientalis

XML Treatment for Torrenticola (Torrenticola) neodentifera

XML Treatment for Hygrobates (Rivobates) cf. microepimeratus

XML Treatment for Atractides (Atractides) constrictus

XML Treatment for Atractides (Atractides) gracilis

XML Treatment for Atractides (Atractides) ermilovi

XML Treatment for Feltria (Feltria) kuluensis
